# Comparing CABG and PCI across the globe based on current regional registry evidence

**DOI:** 10.1038/s41598-022-25853-4

**Published:** 2022-12-22

**Authors:** Tulio Caldonazo, Hristo Kirov, Leoni Lu Riedel, Mario Gaudino, Torsten Doenst

**Affiliations:** 1 Department of Cardiothoracic Surgery, Jena University Hospital, University of Jena, Am Klinikum 1, 07747 Jena, Germany; 2 Department of Cardiothoracic Surgery at New York Presbyterian, Weill Cornell Medical Center, New York, USA

**Keywords:** Interventional cardiology, Health policy

## Abstract

There is an ongoing debate whether coronary artery bypass grafting (CABG) or percutaneous coronary intervention (PCI) provide better results for the treatment of coronary artery disease (CAD). We aimed to evaluate the impact of CABG or PCI on long-term survival based on local reports from different regions in the world. We systematically searched MEDLINE selecting studies that compared outcomes for CABG or PCI as a treatment for CAD in the last 10 years. Reports without all-cause mortality, multi-national cohorts, hybrid revascularization populations were excluded. Qualifying studies were statistically compared, and their geographic location visualized on a world map. From 5126 studies, one randomized and twenty-two observational studies (19 risk-adjusted) met the inclusion criteria. The mean follow-up was 4.7 ± 7 years and 18 different countries were included. In 17 studies, CABG was associated with better survival during follow-up, six studies showed no significant difference, and no study favored PCI. Periprocedural mortality was not different in seven, lower with PCI in one, lower with CABG in three and not reported in 12 studies. In regional registry-type comparisons, CABG is associated with better long-term survival compared to PCI in most regions of the world without evidence for higher periprocedural mortality.

## Introduction

There is an ongoing debate whether percutaneous coronary intervention (PCI) or coronary artery bypass grafting (CABG) provide better results for the treatment of coronary artery disease (CAD)^1–3^. A recent patient-level meta-analysis of all prospective randomized trials performed between 1996 and 2019 demonstrated a significant survival advantage for CABG after 5 years^4^, which appears to be greatest if anatomical complexity of CAD is high. Guidelines specifically outline when to recommend which treatment option^5^.

While randomized clinical trials (RCT) have been widely accepted as the gold standard for assessing the efficacy of different treatment options, incl. interventions^6,7^, the outcomes are bound to reflect the average treatment effect for an often selected patient population^8^. For instance, it is well known that multicentre RCTs often have outcome differences between different participating centres^9^.

In contrast, registry data, although heavily burdened with various biases (incl. selection or treatment allocation, surgeon/interventionalist, publication or investigator biases), may be considered to reflect the regional outcomes for a large fraction of the affected patient population in that region and therefore may provide information on the results of the available treatment modalities within the individual regions^10^. This information is not provided by randomized trials and the data from these registries may even provide external validation of outcomes of RCTs^8,10,11^.

We therefore analyzed the results of all local studies that compared long-term survival after CABG or PCI in localizable regions of the world in the last 10 years. We selected this recent time frame to limit the influence of older technology and older medical therapy.

## Methods

Ethical approval of this analysis was not required as no human or animal subjects were involved.

### Search strategy

We performed a comprehensive literature search to identify contemporary studies reporting long-term mortality from populations that received CABG or PCI as a treatment for coronary disease. Searches were run on July 17, 2021 in the Ovid MEDLINE^®^ database. The search strategy is available in Supplementary Table 1.

### Study selection and data extraction

The study selection followed the Preferred Reporting Items for Systematic Reviews and Meta-Analyses (PRISMA) strategy. After de-duplication, records were screened by two independent reviewers (TC and HK). Any discrepancies and disagreements were resolved by a third author (TD). Titles and abstracts were reviewed against pre-defined inclusion and exclusion criteria. Studies were considered for inclusion if they were written in English and reported direct comparison between populations that received CABG or PCI. Reports without all-cause mortality as an endpoint, multinational cohorts, hybrid revascularization populations and duplicates were excluded. Faced with studies of the same center/region, the largest and most recent series were included. Also in this context, animal studies, abstracts, case reports, commentaries, editorials, expert opinions, conference presentations were excluded.

The full text was pulled for the selected studies for a second round of eligibility screening. References for articles selected were also reviewed for relevant studies not captured by the original search.

The quality of the included studies was assessed using the Newcastle–Ottawa Scale for observational studies (Supplementary Table 2) and the Cochrane risk-of-bias tool for randomized trials (Supplementary Table 3)^12^.

Two reviewers (TC and HK) independently performed data extraction, and the accuracy was verified by a third author (TD). The variables included were: study characteristics (publication year, country, sample size, and mean follow-up) and patient demographics (age, sex, left ventricular ejection fraction [LVEF], hypertension, diabetes mellitus, smoking status, prior cerebrovascular accident [CVA], prior myocardial infarction [MI], and prior percutaneous coronary intervention [PCI]).

The individual mortality rates of each group (CABG and PCI) were extracted per study and were integrated to an overall mean by a weighted average using the population size as a basis. The geographic location from the qualifying studies was visualized by locating the study sites on a world map. In addition, we screened the selected studies for information on periprocedural outcomes and used the available randomized evidence for comparison of the prevalent operative risk (i.e., 30-day/in-hospital mortality).

## Results

**Figure 1:**
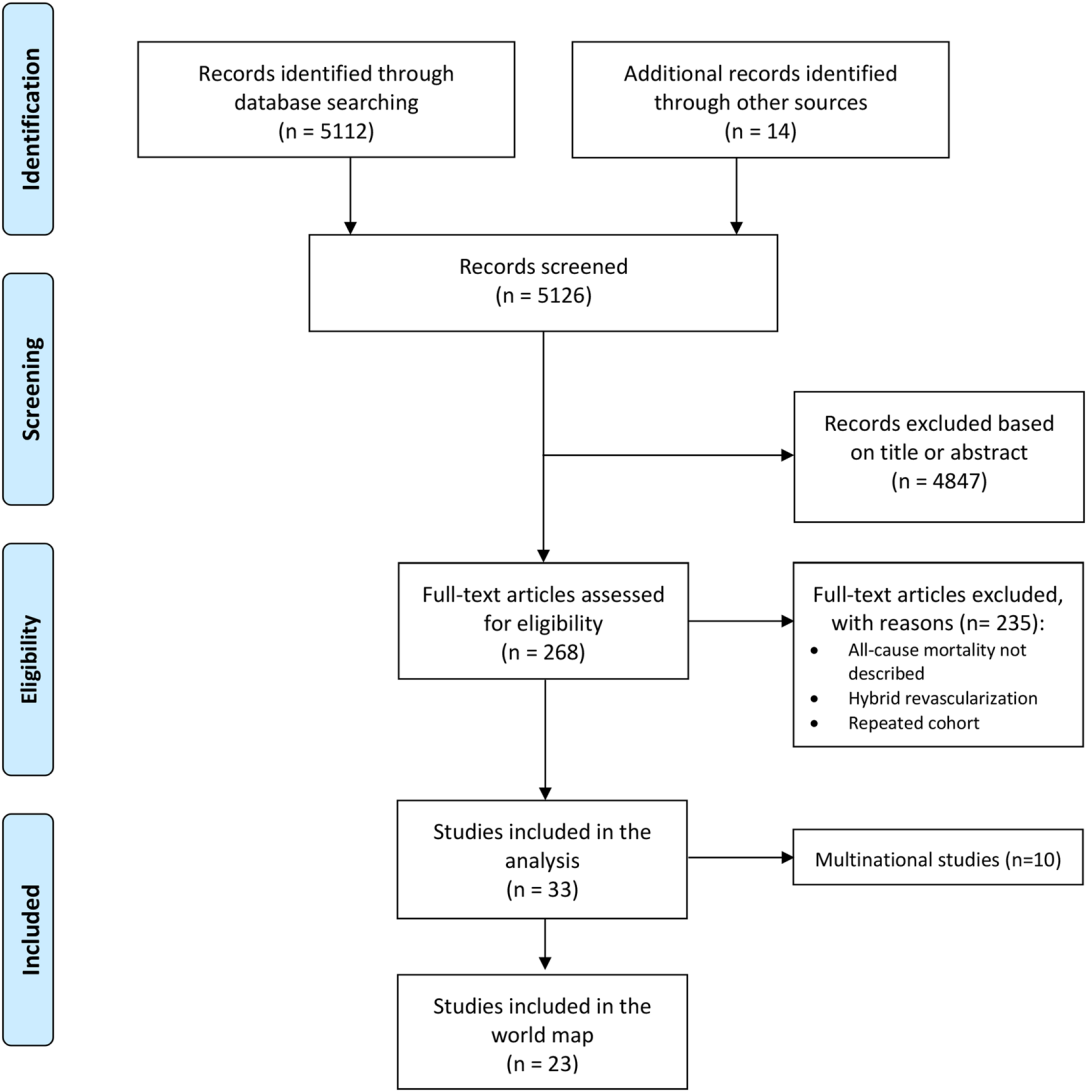
PRISMA diagram describing the systematic research, inclusion/exclusion criteria and the study structure.

Figure 1 shows the PRISMA flowchart for study selection. A total of 5126 studies were retrieved from the systematic search, of which 23 met the criteria for inclusion in the final analysis.

Table 1 provides the details of the included studies. The included studies were published between 2010 and 2021, one study presented a randomized population^13^ and the others were observational cohort studies. Five originated from different regions in the United States, two from different regions in Canada, and one each from Brazil, Poland, Taiwan, Australia, Italy, Finland, Norway, Japan, China, Sweden, Germany, Netherlands, Israel, Great Britain, Iran and South Korea. From all the observational studies, nineteen were based on risk-adjusted populations. A total of 186,696 patients were included in the final analysis, and the number of patients in each study ranged from 173 to 73,730. The number of patients in the risk-adjusted populations was 73,272 (31,911 CABG and 41,361 PCI).

**Table 1:** Summary of included studies (references are reported in the Supplementary Material).

Author	Year	Country/region	No of patients	Mean follow-up (y)	Population comparability	Endpoint long-term mortality
Hueb (MASS-II)	2010	Brazil	611	10	RCT	No difference
Zalweska-Adamiec	2013	Poland	257	1.3	Not adjusted	No difference
Chou	2014	Taiwan	1287	5.4	MR	Favors CABG
Sugumar	2014	Australia	8970	3.1	PSM	Favors CABG
Bangalore	2015	New York State, United States	34,819	2.9	PSM	No difference
Krishnaswami	2015	San Francisco Bay Area, United States	1015	5	MR	No difference
Nicolini	2015	Italy	1388	5.5 (CABG) 4.4 (PCI)	PSM	Favors CABG
Lautamäki	2016	Finland	268	2.1	PSM	Favors CABG
Mølstad	2016	Norway	22,880	8	MR	No difference
Yamaji	2016	Japan	5651	10.1 (CABG) 9.9 (PCI)	MR	Favors CABG
Zheng	2016	China	4046	3	PSM	Favors CABG
Nyström	2017	Sweden	2546	8	PSM	Favors CABG
Roberts	2017	North Carolina, United States	4687	5.1	PSM	Favors CABG
Iribarne	2018	New England, United States	73,730	4.3	PSM	Favors CABG
Merkle	2018	Germany	561	6	MR	Favors CABG
Milojevic	2018	Netherlands	1897	39 (CABG) 33 (PCI)	Not adjusted	Favors CABG
Nagendran	2018	Alberta, Canada	2837	5.5	PSM	Favors CABG
Ram	2018	Israel	1063	3	MR	Favors CABG
Shah	2018	Great Britain	717	5	PSM	Favors CABG
Khosravi	2019	Iran	173	0.5	Not adjusted	No difference
Lee	2020	South Korea	2240	10	PSM	Favors CABG
Tam	2020	Ontario, Canada	14,235	5.5	PSM	Favors CABG
Huckaby	2021	Pennsylvania, United States	1091	4 (CABG) 3.5 (PCI)	PSM	Favors CABG

*CABG* coronary artery bypass grafting, *MR* multivariable regression, *PCI* percutaneous coronary intervention, *PSM* propensity score matching, *RCT* randomized clinical trial.

Tables 2, 3, 4 summarizes the demographic data of the patient population in each study. The mean age ranged from 53.0 to 75.2 years in the CABG group and 56.9 to 74.0 in the PCI group. Percentages of male patients ranged from 56.9 to 87.9 in the CABG group and 56.9 to 89.5 in the PCI group. The prevalence of hypertension ranged from 21.6 to 90.8% in the CABG group and 32.5 to 93.7% in the PCI group. The prevalence of diabetes mellitus ranged from 8.6 to 75.9% in the CABG group and 11.6 to 74.6% in the PCI group. From the 23 studies, just 7 reported patients with not exclusively elective cases. The percentage of not elective cases was in all studies similar in the CABG and in the PCI group.

**Table 2:** Demographic data from the included studies (part 1).

Author	Age (Mean ± SD)		Male (%)		Mean LVEF (mean ± SD)		HP (%)		DM (%)		Smoking (%)		Prior CVA (%)		Prior MI (%)		Prior PCI (%)	
	CABG	PCI	CABG	PCI	CABG	PCI	CABG	PCI	CABG	PCI	CABG	PCI	CABG	PCI	CABG	PCI	CABG	PCI
Hueb (MASS II), 2010	60 ± 9	60 ± 9	72	67	67 ± 9	67 ± 8	63	61	29	23	32	27	NR	NR	41	52	0	0
Zalweska-Adamiec, 2013	66 ± 10.2	62 ± 13.7	72.8	89.5	51 ± 10.9	52 ± 9.5	76.9	68.4	25.4	21.1	54.6	72.3	NR	NR	41.4	26.3	NR	NR
Chou, 2014	NR	NR	65.7	55.5	NR	NR	82.5	82.4	75.9	74.6	NR	NR	NR	NR	NR	NR	NR	NR
Sugumar, 2014 GFR > 60	64.9 ± 10.9	63.6 ± 11.3	73.5	78.9	NR	NR	68.4	67.2	25	23.8	18.9	22.7	NR	NR	7.3	19.8	NR	NR
Sugumar, 2014 GFR 30–59	75.2 ± 9	73.7 ± 8.6	56.9	61.1	NR	NR	82.1	81.4	36.3	36.3	9.5	9.3	NR	NR	20.8	29.7	NR	NR
Sugumar, 2014 GFR < 30	68.7 ± 11.7	69.3 ± 11.4	63.5	70.3	NR	NR	83.8	86.5	33.8	46	14.9	16.2	NR	NR	35.1	46	NR	NR
Bangalore, 2015	65.3 ± 10.6	65 ± 11.2	74.2	70.8	NR	NR	NR	NR	NR	NR	NR	NR	NR	NR	53	65.8	18	31.7
Krishnaswami, 2015	63.4 ± 9.3	64.7 ± 10.6	64.8	60.1	NR	NR	90.8	93.7	76.7	71	45.3	43.6	6.9	7.2	41	43.1	NR	NR
Nicolini, 2015	NR	NR	66	56.9	NR	NR	83.7	82.7	19.7	22.2	4.5	5.5	NR	NR	47.2	27.1	NR	NR
Lautamäki, 2016	70.7 ± 9.9	73.1 ± 9.9	58.8	56.4	NR	NR	75.7	84.5	44.6	52.7	NR	NR	10.8	13.6	47.3	20	16.2	19.1

*CABG* coronary arterial bypass graft, *CVA* cerebrovascular accident, *DM* diabetes, *GFR* glomerular filtration rate, *HP* hypertension, *LVEF* left ventricular ejection fraction, *MI* myocardial infarction, *NR* not reported, *PCI* percutaneous coronary intervention, *SD* standard deviation.

**Table 3:** Demographic data from the included studies (part 2).

Author	Age (mean ± SD)		Male (%)		Mean LVEF (mean ± SD)		HP (%)		DM (%)		Smoking (%)		Prior CVA (%)		Prior MI (%)		Prior PCI (%)	
	CABG	PCI	CABG	PCI	CABG	PCI	CABG	PCI	CABG	PCI	CABG	PCI	CABG	PCI	CABG	PCI	CABG	PCI
Mølstad, 2016	67 ± 10	65 ± 11	78.2	73	67 ± 12	68 ± 12	36.5	32.5	16.4	13.3	19.4	21.6	NR	NR	36.5	36.3	8.4	17
Yamaji, 2016	67.5 ± 9	69.4 ± 10	72	70	58.2 ± 14.6	59.4 ± 14	77	82	36	36	25	25	27	19	32	23	NR	NR
Zheng, 2016	62.2 ± 9.1	59.9 ± 10.7	82	78.6	60.2 ± 8.2	63.1 ± 7.2	64.3	54.2	31	24.1	53.6	46.5	NR	NR	38.1	23.6	9.7	22.5
Nyström, 2017	57.2 ± 10	61.1 ± 10.5	63.4	58.9	NR	NR	NR	NR	NR	NR	15	14.5	6.6	9.6	54.3	47.6	NR	NR
Roberts, 2017 PCI DES	64 ± 4	62 ± 4	72	68.1	NR	NR	70.7	67.6	33.4	30	43.3	40.8	10.2	5.9	41.8	46.8	NR	NR
Iribarne, 2018	NR	NR	79.5	66	NR	NR	NR	NR	48.2	38.9	30.2	28.7	NR	NR	25.8	25.8	NR	NR
Merkle, 2018	64 ± 4.7	67 ± 3.5	84	78	NR	NR	86.8	73.7	29.2	17.2	23.6	20.2	NR	NR	NR	NR	NR	NR
Milojevic, 2018	53 ± 2.7	56.9 ± 3	87.9	79.9	NR	NR	21.6	40.5	8.6	11.6	57.8	58	NR	NR	NR	NR	NR	NR
Nagendran, 2018	65.6 ± 9.5	64.6 ± 11.4	80	75	NR	NR	85	79	NR	NR	19	19	NR	NR	14	43	4	4

*CABG* coronary arterial bypass graft, *CVA* cerebrovascular accident, *DES* drug eluting stents, *DM* diabetes, *HP* hypertension, *LVEF* left ventricular ejection fraction, *MI* myocardial infarction, *NR* not reported, *PCI* percutaneous coronary intervention, *SD* standard deviation.

**Table 4:** Demographic data from the included studies (part 3).

Author	Age (MEAN ± SD)		Male (%)		Mean LVEF (mean ± SD)		HP (%)		DM (%)		Smoking (%)		Prior CVA (%)		Prior MI (%)		Prior PCI (%)	
	CABG	PCI	CABG	PCI	CABG	PCI	CABG	PCI	CABG	PCI	CABG	PCI	CABG	PCI	CABG	PCI	CABG	PCI
Ram, 2018	66 ± 10	70 ± 12	81	75	NR	NR	76	82	45	43	25	22	11	3	NR	NR	27	47
Shah, 2018	65.2 ± 12.9	72.1 ± 11 ±	85.9	82.2	NR	NR	72.3	77.2	32.9	30.1	19.1	26.5	NR	NR	83.7	55.7	6.8	24.2
Khosravi, 2019	63.8 ± 9.2	63.8 ± 11.4	75.6	79.3	NR	NR	40	38.6	31.4	32.2	11.8	14.1	0	0	2.3	1.2	NR	NR
Lee, 2020	62. ± 9.4	61.3 ± 11.7	72.9	70.7	NR	NR	49.4	49.5	23.5	22.9	29.8	25.6	7.3	7.1	11.6	8.1	11	18.1
Tam, 2020	65.7 ± 9.4	68.3 ± 11.2	77.9	72.5	NR	NR	80.5	83.3	NR	NR	19.3	16.3	NR	NR	23.5	27.4	NR	NR
Huckaby, 2021	74 ± 2.2	74 ± 3.2	64.6	65	53 ± 4	55 ± 4.5	87.9	88	41.9	41	15.8	17.2	NR	NR	47.4	46.3	NR	NR

*CABG* coronary arterial bypass graft, *CVA* cerebrovascular accident, *DM* diabetes, *HP* hypertension, *LVEF* left ventricular ejection fraction, *MI* myocardial infarction, *NR* not reported, *PCI* percutaneous coronary intervention, *SD* standard deviation.

**Figure 2:**
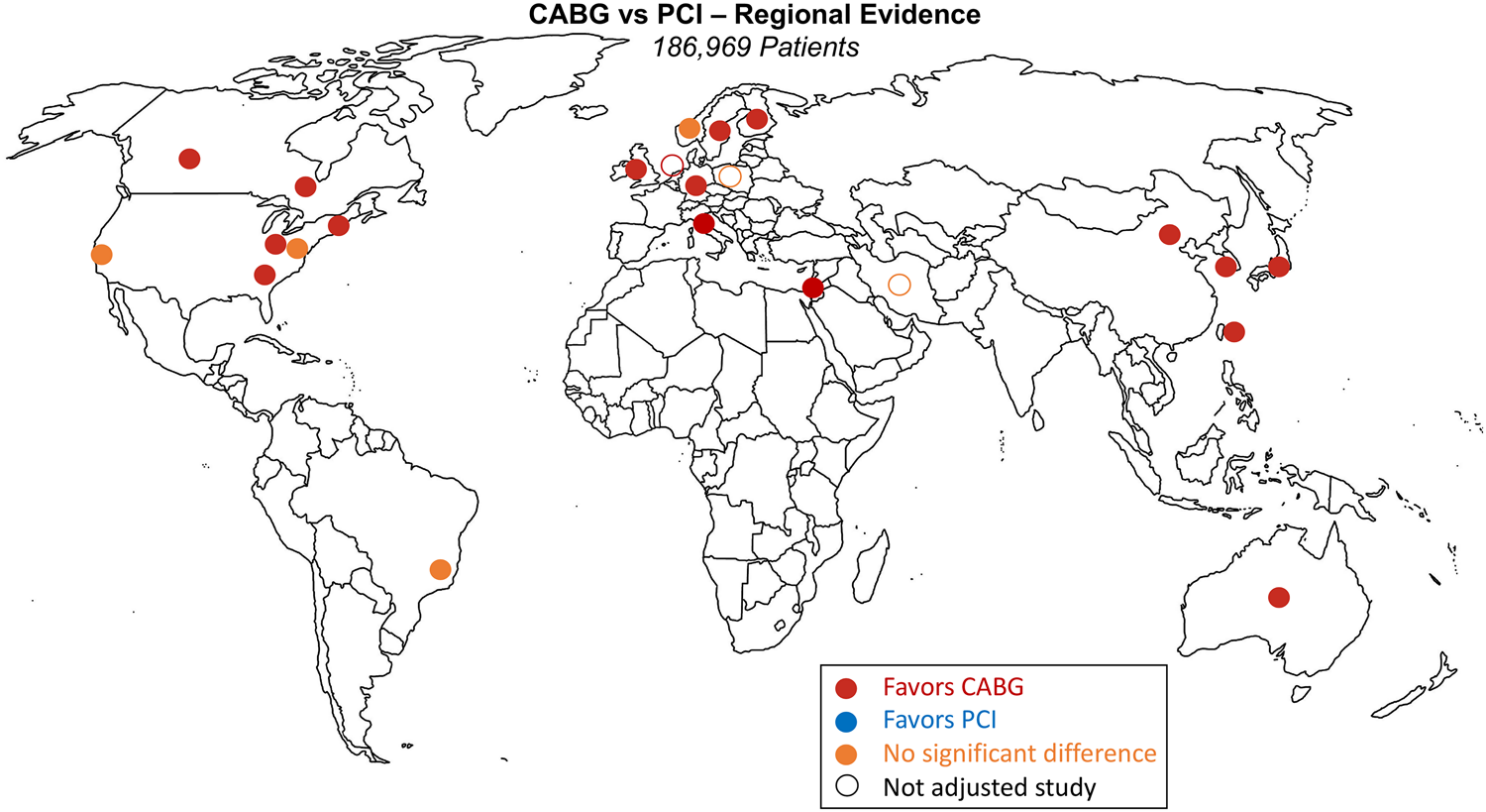
(Central Illustration). World map locating the study sites and the corresponding outcome for the endpoint long-term all-cause mortality.

Figure 2 (Central Illustration) shows a world map locating the study sites and the corresponding outcome for the endpoint of long-term all-cause mortality. In 17 studies, CABG was associated with better survival during follow-up, six studies, incl. the MASS II randomized trial from Brazil^13^, showed no significant difference, and no study favored PCI. Non-risk-adjusted studies are marked with an open circle.

**Figure 3:**
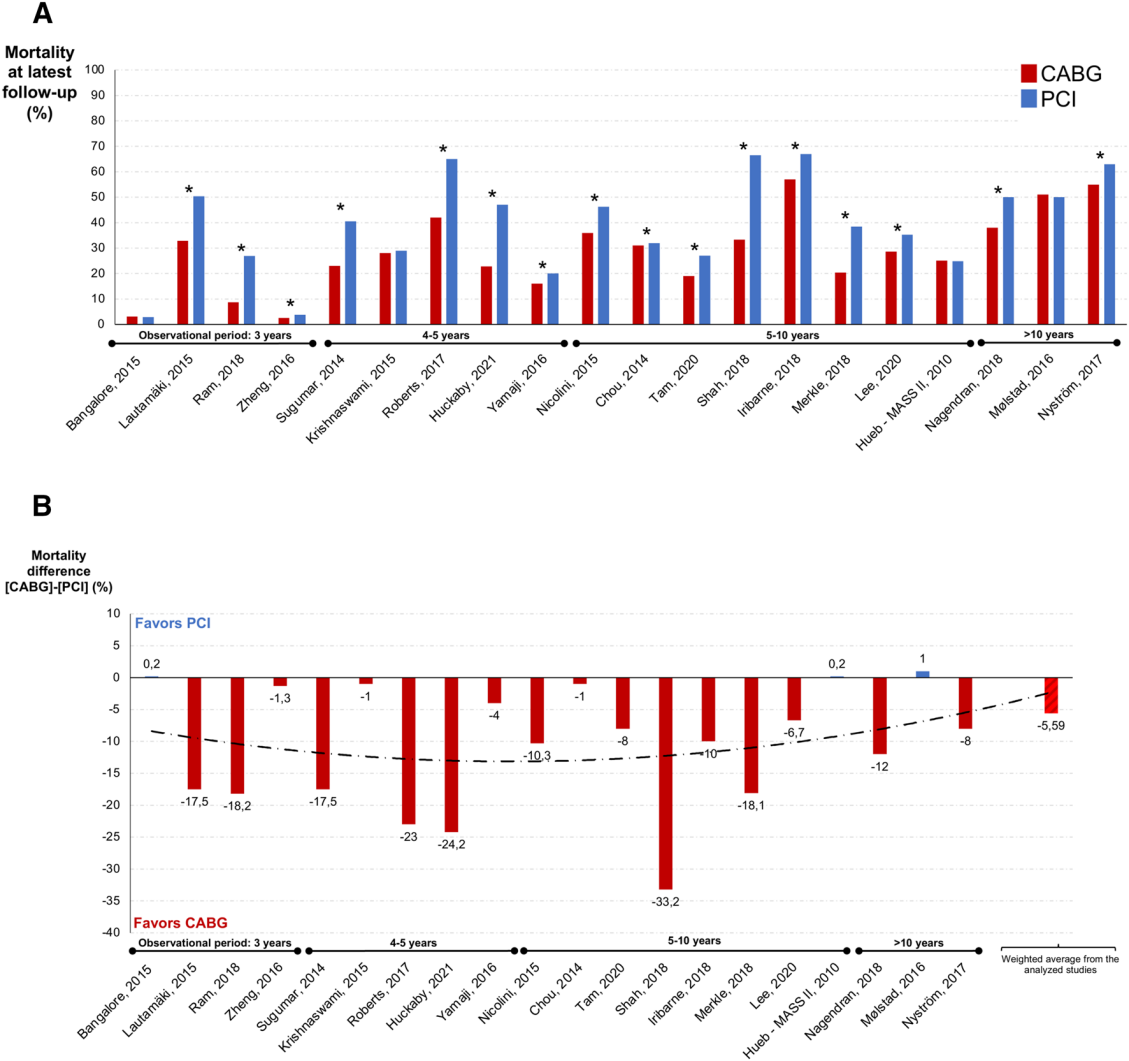
Actual mortality rates (**A**) and difference of the mortality rates in percentage (**B**) of the analyzed studies for PCI and CABG (excluding the non-risk-adjusted ones).

Figure 3 shows the actual mortality rates for CABG and PCI of the analyzed studies at latest follow-up (A) and the resulting mortality difference at the end of the reported observation period (B). The mortality difference between CABG and PCI ranged from − 33.2^14^ to 1.0%^15^. The average mortality difference between CABG and PCI (weighted for the number of patients in each adjusted study) was − 5.59%. The four risk-adjusted studies showing no significant difference between the two invasive treatment options came from the United States—New York^16^ and San Francisco^17^—from Brazil^13^ and from Norway^15^ with a follow-up of 2.9, 5, 10 and 16 years, respectively.

**Figure 4:**
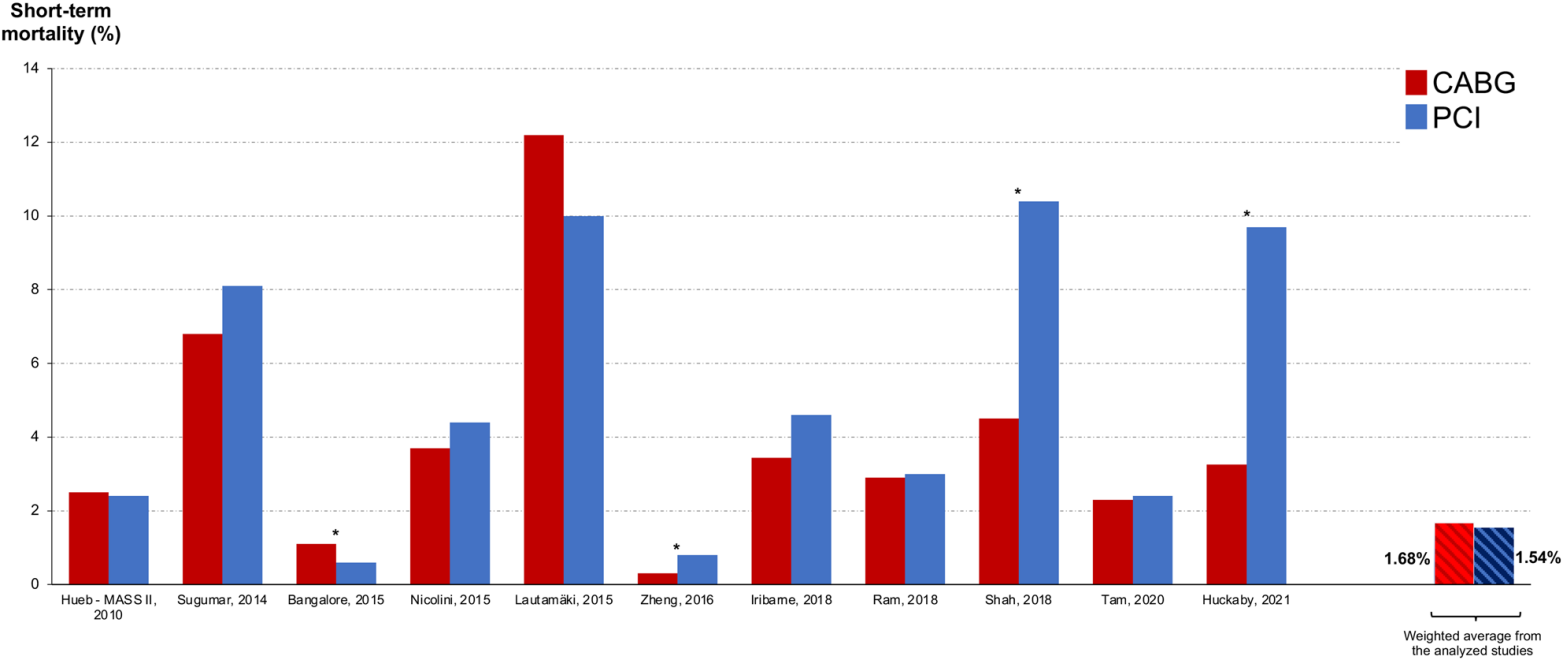
Periprocedural mortality found in the analyzed studies for PCI and CABG (excluding the non-risk-adjusted ones).

Table 5 lists the characteristics reported for periprocedural mortality from the selected studies. From the 23 studies, 11 reported information on periprocedural mortality. The majority presented 30-day mortality. Figure 4 shows the actual values reported for CABG and PCI in the analyzed studies. Periprocedural mortality rates were significantly lower in the PCI group in only one study. In three studies, periprocedural mortality rates were lower in the CABG group and 7 studies showed no significant difference between the two techniques (including the randomized trial). The remaining 12 studies did not report peri-procedural mortality. Excluding the non-risk-adjusted studies and weighted the results based on the included patient number in each study revealed short-term mortality rates of 1.68% and 1.54% for CABG and PCI, respectively.

**Table 5:** Periprocedural mortality data from the selected studies.

Author	Definition	Outcome
Hueb (MASS II)	In-hospital	No statistical difference between the groups
Zalweska-Adamiec	NR	NR
Chou	NR	NR
Sugumar	30-day	No statistical difference between the groups
Bangalore	In-hospital or 30-day	Favors PCI
Krishnaswami	NR	NR
Nicolini	30-day	No statistical difference between the groups
Lautamäki	30-day	No statistical difference between the groups
Mølstad	NR	NR
Yamaji	NR	NR
Zheng	30-day	Favors CABG
Nyström	NR	NR
Roberts	NR	NR
Iribarne	30-day	No statistical difference between the groups
Merkle	NR	NR
Milojevic	NR	NR
Nagendran	NR	NR
Ram	30-day	No statistical difference between the groups
Shah	In-hospital	Favors CABG
Khosravi	NR	NR
Lee	NR	NR
Tam	In-hospital or 30-day	No statistical difference between the groups
Huckaby	30-day	Favors CABG

*CABG* coronary artery bypass grafting, *PCI* percutaneous coronary intervention, *NR* not reported.

## Discussion

In this analysis we show that in regional registry-type comparisons, CABG is associated with better long-term survival compared to PCI in most regions of the world without evidence for higher periprocedural mortality. This information may assist decision-making for invasive treatment of CAD.

Based on our analysis, CABG appears to provide a survival advantage for most patients in most regions of the world if compared to PCI based on registry data. The available randomized mostly multi-national evidence has been less clear (possibly due to the limited patient number in most trials) and only the combination of 11 randomized trials resulted in the demonstration of a 2% total survival advantage of CABG over PCI after 5 years. Since many subgroup analyses did not find such differences^4,18,19^ and the ISCHEMIA trial renewed concern on survival effects from treating inducible ischemia, there is currently a strong debate about the resulting treatment recommendation^1^. The discussion has culminated by the downgrading of treatment recommendations for CABG to improve survival from Class I to Class IIb in the most recent guidelines of the AHA/ACC^20^, which led to heavy criticism from American and international surgical societies^21^. In light of this discussion, it is impressive to observe the striking differences we show here when examining the local outcomes of propensity matched all-comers registries from all over the world. The average survival difference was almost three times that in the randomized meta-analysis (5.59% vs 2%). One may wonder why some randomized trials have been unable to reproduce this effect or why registries often show so much difference.

Possible explanations may be manifold. One explanation may be a high degree of patient selection in randomized clinical trials. EuroSCORE II and perioperative mortality in trials are usually around 1–2%^4^, while real life surgical data are generally higher^22,23^, suggesting that more complex patients with higher perioperative risks are subjected to surgery in daily practice compared to those included in randomized trials^24,25^. Inspecting the individual outcomes in Fig. 4 supports this impression, with 30 days mortality rates of the individual studies ranging as high as 12%. Nevertheless, the average periprocedural mortality weighted for the numbers of patients included is lower and closer to the randomized populations, making a selection bias also possible in the propensity matched registry populations. Inspecting the registry studies in detail, reveals very low mortality in two registries that contain large patient numbers. It is not clear why mortality in these studies is so low, but the difference between the national registry reports^22,23^ and the summary of all individual local reports in this analysis supports the suspicion for a certain degree of selection in those regions or selection and/or publication bias.

It is interesting to note in this context, that the largest registry with the lowest periprocedural mortality is the only one also reporting a significantly lower periprocedural mortality compared to CABG^16^. The same data source, the New York State database, was used for a very similar publication only 7 years earlier on over 17,000 patients^26^. This analysis did not find periprocedural differences in mortality and supported the better survival with CABG in the long run^26^. Since it is unlikely to expect such substantial improvements in PCI in only 7 years, one may suspect that more selection may have taken place in the more recent publication. Indeed, inspecting the exclusion criteria of the two studies reveals a much longer list in the more recent study^16^. Specifically, the recent analysis excluded all PCI procedures not using everolimus-eluting stents^16^, a significant selection bias is possible. It is interesting to read a recent publication from Pittsburg in this context, which may be affected by a similar selection bias but in “the other direction”. The authors selected a CABG population of only fully arterial bypass grafting and performed a propensity matched comparison to PCI based on all PCI patients in that center^27^. They demonstrated reductions of mortality and MACCE of about 50% with CABG over a 5-year median observation period.

These and other biases prevent registry data from being the ideal source for generalized treatment recommendations. Aside from the problems of appropriate risk-adjustment, local factors have been discussed as serious concern^9^. A patient in Europe may have a different outcome after medical or invasive therapy than the same patient in Asia or America. Our assessment of all local registries that report risk-adjusted comparison between CABG and PCI in the world addresses this issue and shows an impressive trend for the value of CABG. Thus, regional differences in patient characteristics may be overrated because the CABG treatment effect is visible worldwide.

This globally apparent observation raises the question of the underlying mechanism. We had suggested with our concept of surgical collateralization that any life-prolonging (i.e., prognostic) effect in the invasive treatment of chronic coronary syndrome appears to be due to prevention of future myocardial infarctions^3^. This concept has the potential to explain the perceived inconsistency of the data. We here demonstrate that periprocedural risk in registries is usually higher in most regions of the world than in randomized populations (Fig. 4)^4^. In the low-risk randomized populations, the differences between CABG and PCI are not massive, but CABG always comes out superior in subgroups when the rates of myocardial infarctions are lower than with PCI^28,29^. Patients in daily practice may carry higher risks of myocardial infarction than in randomized trials, which may explain why the risk-adjusted registry comparisons are in favor of CABG for long-term-outcome. Importantly, a lower rate of myocardial infarctions in the CABG population was present in all studies that reported the rates (including the largest by Bangalore et al.^16^).

Additionally, the distal anastomosis tissue manipulation during CABG may result in surgical denervation, which might have an afferent component (reducing pain sensation) and an efferent component (reducing alpha-adrenergic coronary vasoconstriction). Both factors, combined with the protection from future myocardial infarctions through surgical collateralization may possibly contribute to the superiority of CABG over PCI not only in survival but potentially also in long-term quality of life^30–32^.

Irrespective of mechanisms, the most striking finding of our analysis here may be another observation. It is our current understanding that we assess surgical risk and decide between CABG and PCI if risk is low^5^. This guideline-recommended behavior suggests that patients with higher risk are automatically referred to PCI for its naturally presumed lower peri-procedural risk (i.e., mortality). It is therefore striking to note that periprocedural mortality was not different between PCI and CABG in the available data from the observational studies. We had addressed issues with risk–benefit evaluations before^33^ and eluted to the lack of differences in 30-day mortality in the randomized populations from the patient-level meta-analysis by Head et al.^4,33^. We now show the same for daily-risk registry populations. This information may be hard to digest, but it may require us to reconsider our current way of thinking and acting. There is no doubt that PCI is much less invasive and associated with faster mobilization, less pain and a few weeks faster improvement in quality of life^34–36^. However, PCI does not appear to be less dangerous than CABG considering mortality as endpoint.

In other words, CABG appears to be much less dangerous than expected not only in randomized populations but also in daily practice. The surprisingly low 30-day mortality (in comparison to PCI) may possibly be explained to some extent through the improvement in perioperative care, such as the reduction of cardiotoxic medications, optimization of anesthetic methods and the exploitation of preconditioning mechanisms, contributing to cardioprotection^37,38^ and potentially reducing other perioperative risks.

Considering this observation would require us to compare short-term outcomes between CABG and PCI at higher risk groups, because patients with higher periprocedural risks may also have higher risks for myocardial infarction, so that CABG may turn out to be the preferred treatment strategy in many of these patients. The most important challenge in this context appears to be a better characterization of future risks of myocardial infarctions.

As previously stated, registry data are considered to reflect the regional treatment effect for the majority of patients in that region. Their value has been considered as high for the purpose of external validation of RCTs performed in the field. The information we provide with our analysis would only serve the external validation aspect well, if one accepts the outcomes of the patient-level meta-analysis^4^. However, the many (underpowered) sub-analyses and the suggested mechanistic approach to invasive CAD treatment^39^ suggest an individualized approach which should take short-term risks and long-term benefits in the local environment into consideration. We provide important information here for this discussion.

## Conclusion

We demonstrate that in regional registry-type comparisons, CABG is associated with better long-term survival compared to PCI in most regions of the world without evidence for higher periprocedural mortality. This information may assist decision-making for invasive treatment of CAD.

## Supplementary Information

Supplementary Tables.

## Data Availability

The data underlying this article are available in the article and in its online supplementary material.

## Abbreviations

CABG: Coronary artery by-pass grafting
CAD: Coronary artery disease
PCI: Percutaneous coronary intervention
PRISMA: Preferred Reporting Items for Systematic Reviews and Meta-Analyses
RCT: Randomized clinical trials
