# Supplementary material for: Comparing CABG and PCI across the globe based on current regional registry evidence
Source: Sci Rep. 2022 Dec 22;12:22164. doi: 10.1038/s41598-022-25853-4 (PMC9780238; doi:10.1038/s41598-022-25853-4)
Supplement: Supplementary file 1 — Supplementary Tables. [file 41598_2022_25853_MOESM1_ESM.docx]

**Supplementary Material**

**Supplementary Table 1**: Search strategy for Ovid MEDLINE.

**Supplementary Table 2:** Assessment of risk of bias using the Newcastle-Ottawa Scale.

**Supplementary Table 3**: Assessment of risk of bias using the Cochrane Risk of Bias Tool.

**Supplementary Table 4**: Number of target vessels.

**Supplementary References**

Supplementary Table 1. Search strategy for Ovid MEDLINE.

| Search: ((Percutaneous coronary intervention) AND (Coronary artery bypass grafting)) AND (("2011/01/01"[Date - Publication] : "2021/07/17"[Date - Publication])), Filters: English |
| --- |
| (("percutaneous coronary intervention"[MeSH Terms] OR ("percutaneous"[All Fields] AND "coronary"[All Fields] AND "intervention"[All Fields]) OR "percutaneous coronary intervention"[All Fields]) AND ("coronary artery bypass"[MeSH Terms] OR ("coronary"[All Fields] AND "artery"[All Fields] AND "bypass"[All Fields]) OR "coronary artery bypass"[All Fields] OR ("coronary"[All Fields] AND "artery"[All Fields] AND "bypass"[All Fields] AND "grafting"[All Fields]) OR "coronary artery bypass grafting"[All Fields]) AND 2011/01/01:2021/07/17[Date - Publication]) AND (english[Filter]) |
| **Translations**  **Percutaneous coronary intervention:** "percutaneous coronary intervention"[MeSH Terms] OR ("percutaneous"[All Fields] AND "coronary"[All Fields] AND "intervention"[All Fields]) OR "percutaneous coronary intervention"[All Fields]  **Coronary artery bypass grafting:** "coronary artery bypass"[MeSH Terms] OR ("coronary"[All Fields] AND "artery"[All Fields] AND "bypass"[All Fields]) OR "coronary artery bypass"[All Fields] OR ("coronary"[All Fields] AND "artery"[All Fields] AND "bypass"[All Fields] AND "grafting"[All Fields]) OR "coronary artery bypass grafting"[All Fields] |

Supplementary Table 2. Assessment of risk of bias using the Newcastle-Ottawa Scale.

| AUTHOR | SELECTION | COMPARABILITY | OUTCOME/ EXPOSURE |
| --- | --- | --- | --- |
| Zalweska-Adamiec, 2013 (1) | **** | * | *** |
| Chou, 2014 (2) | **** | ** | *** |
| Sugumar, 2014 (3) | **** | ** | *** |
| Bangalore, 2015 (4) | **** | ** | *** |
| Krishnaswami, 2015 (5) | **** | ** | *** |
| Nicolini, 2015 (6) | **** | ** | *** |
| Lautamäki, 2016 (7) | **** | ** | *** |
| Mølstad, 2016 (8) | **** | ** | *** |
| Yamaji, 2016 (9) | **** | ** | *** |
| Zheng, 2016 (10) | **** | ** | *** |
| Nyström, 2017 (11) | **** | ** | *** |
| Roberts, 2017 (12) | **** | ** | *** |
| Iribarne, 2018 (13) | **** | ** | *** |
| Merkle, 2018 (14) | **** | ** | *** |
| Milojevic, 2018 (15) | **** | * | *** |
| Nagendran, 2018 (16) | **** | ** | *** |
| Ram, 2018 (17) | **** | ** | *** |
| Shah, 2018 (18) | **** | ** | *** |
| Khosravi, 2019 (19) | ******** | ***** | ******* |
| Lee, 2020 (20) | ******** | ****** | ******* |
| Tam, 2020 (21) | ******** | ****** | ******* |
| Huckaby, 2021 (22) | ******** | ****** | ******* |

**Supplementary Table 3**: Assessment of risk of bias using the Cochrane Risk of Bias Tool.

| **Author** | **Year of publication** | **RANDOM SEQUENCE GENERATION** | **ALLOCATION CONCEALMENT** | **BLINDING OF PARTICIPANTS** | **BLINDING OF**  **OUTCOME ASSESSMENT** | **INCOMPLETE**  **OUTCOME DATA** | **SELECTIVE**  **REPORTING** | **OTHER SOURCES**  **OF BIAS** |  |
| --- | --- | --- | --- | --- | --- | --- | --- | --- | --- |
| Hueb, MASS II (23) | | 2004 |  |  |  |  |  |  |  |
|  | | | | + | Low Risk | | | |  |
|  |  |  |  | ? | Uncertain | | | |  |
|  |  |  |  | - | High Risk | | | |  |

**Supplementary Table 4**: Number of target vessels.

| **Author Name** | **Mean Cross Clamp Time (minutes)** | **Number of Target Vessels (% or Mean±SD)** | | | |
| --- | --- | --- | --- | --- | --- |
|  |  | **Double-vessel disease** | | **Triple-vessel disease** | |
|  |  | **CABG** | **PCI** | **CABG** | **PCI** |
| Hueb (MASS-II) | NR | 42 | 42 | 58 | 58 |
| Zalweska-Adamiec |  | 11.8 | 36.8 | 83.5 | 47.4 |
| Chou |  | NR | NR | NR | NR |
| Sugumar |  | 3.3±1.0 | NR | - | - |
| Bangalore |  | 26.3 | 25.9 | 15.3 | 15.2 |
| Krishnaswami |  | 1.3 ±0.68 | 2.6 ±0.98 | - | - |
| Nicolini |  | 17.2 | 14.2 | 18.2 | 17.9 |
| Lautamäki |  | NR | NR | NR | NR |
| Mølstad |  | 17.9 | 30.1 | 79.8 | 23.3 |
| Yamaji |  | NR | NR | NR | NR |
| Zheng |  | 15.8 | 37.1 | 78.8 | 34.5 |
| Nyström |  | NR | NR | NR | NR |
| Roberts |  | 22.6 | 70.7 | 77.4 | 29.3 |
| Iribarne |  | 48.8 | 49.3 | 51.3 | 50.7 |
| Merkle |  | 25.5 | 30.3 | 26.3 | 15.1 |
| Milojevic |  | 30.7 | 23.6 | 42.7 | 11.8 |
| Nagendran |  | NR | NR | NR | NR |
| Ram |  | NR | NR | NR | NR |
| Shah |  | 29.9 | 28.4 | 62.7 | 59.7 |
| Khosravi |  | 22.1 | 8.1 | 64 | 89.7 |
| Lee |  | 26 | 26.3 | 24.8 | 57 |
| Tam |  | 31.4 | 31.7 | 68.6 | 68.3 |
| Huckaby |  | 37.8 | 38.8 | 61.86 | 58.21 |

CABG= coronary artery bypass grafting, PCI= percutaneous coronary intervention, NR= not reported.

**Bibliographic References**

1. Zalewska-Adamiec M, Bachórzewska-Gajewska H, Kralisz P, Nowak K, Hirnle T, Dobrzycki S. Prognosis in patients with left main coronary artery disease managed surgically, percutaneously or medically: a long-term follow-up. Kardiol Pol 2013;71:787-95.

2. Chou CL, Hsieh TC, Wang CH et al. Long-term outcomes of dialysis patients after coronary revascularization: a population-based cohort study in Taiwan. Arch Med Res 2014;45:188-94.

3. Sugumar H, Lancefield TF, Andrianopoulos N et al. Impact of renal function in patients with multi-vessel coronary disease on long-term mortality following coronary artery bypass grafting compared with percutaneous coronary intervention. Int J Cardiol 2014;172:442-9.

4. Bangalore S, Guo Y, Samadashvili Z, Blecker S, Xu J, Hannan EL. Everolimus-Eluting Stents or Bypass Surgery for Multivessel Coronary Disease. New England Journal of Medicine 2015;372:1213-1222.

5. Krishnaswami A, McCulloch CE, Tawadrous M et al. Coronary artery bypass grafting and percutaneous coronary intervention in patients with end-stage renal disease. European Journal of Cardio-Thoracic Surgery 2015;47:e193-e198.

6. Nicolini F, Contini GA, Fortuna D et al. Coronary artery surgery versus percutaneous coronary intervention in octogenarians: long-term results. Ann Thorac Surg 2015;99:567-74.

7. Lautamäki A, Kiviniemi T, Biancari F, Airaksinen J, Juvonen T, Gunn J. Outcome after coronary artery bypass grafting and percutaneous coronary intervention in patients with stage 3b–5 chronic kidney disease. European Journal of Cardio-Thoracic Surgery 2015;49:926-930.

8. Mølstad P, Moer R, Rødevand O. Long-term survival after coronary bypass surgery and percutaneous coronary intervention. Open Heart 2016;3:e000489.

9. Yamaji K, Shiomi H, Morimoto T et al. Effects of Age and Sex on Clinical Outcomes After Percutaneous Coronary Intervention Relative to Coronary Artery Bypass Grafting in Patients With Triple-Vessel Coronary Artery Disease. Circulation 2016;133:1878-1891.

10. Zheng Z, Xu B, Zhang H et al. Coronary Artery Bypass Graft Surgery and Percutaneous Coronary Interventions in Patients With Unprotected Left Main Coronary Artery Disease. JACC Cardiovasc Interv 2016;9:1102-11.

11. Nyström T, Sartipy U, Franzén S et al. PCI Versus CABG in Patients With Type 1 Diabetes and Multivessel Disease. J Am Coll Cardiol 2017;70:1441-1451.

12. Roberts JK, Rao SV, Shaw LK, Gallup DS, Marroquin OC, Patel UD. Comparative Efficacy of Coronary Revascularization Procedures for Multivessel Coronary Artery Disease in Patients With Chronic Kidney Disease. Am J Cardiol 2017;119:1344-1351.

13. Iribarne A, DiScipio AW, Leavitt BJ et al. Comparative effectiveness of coronary artery bypass grafting versus percutaneous coronary intervention in a real-world Surgical Treatment for Ischemic Heart Failure trial population. J Thorac Cardiovasc Surg 2018;156:1410-1421.e2.

14. Merkle J, Zeriouh M, Sabashnikov A et al. Minimally invasive direct coronary artery bypass graft surgery versus percutaneous coronary intervention of the LAD: costs and long-term outcome. Perfusion 2019;34:323-329.

15. Milojevic M, Thuijs DJFM, Head SJ et al. Life-long clinical outcome after the first myocardial revascularization procedures: 40-year follow-up after coronary artery bypass grafting and percutaneous coronary intervention in Rotterdam. Interactive CardioVascular and Thoracic Surgery 2019;28:852-859.

16. Nagendran J, Bozso SJ, Norris CM et al. Coronary Artery Bypass Surgery Improves Outcomes in Patients With Diabetes and Left Ventricular Dysfunction. J Am Coll Cardiol 2018;71:819-827.

17. Ram E, Goldenberg I, Kassif Y et al. Real-life characteristics and outcomes of patients who undergo percutaneous coronary intervention versus coronary artery bypass grafting for left main coronary artery disease: data from the prospective Multi-vessel Coronary Artery Disease (MULTICAD) Israeli Registry†. European Journal of Cardio-Thoracic Surgery 2018;54:717-723.

18. Shah S, Benedetto U, Caputo M, Angelini GD, Vohra HA. Comparison of the survival between coronary artery bypass graft surgery versus percutaneous coronary intervention in patients with poor left ventricular function (ejection fraction &lt;30%): a propensity-matched analysis. European Journal of Cardio-Thoracic Surgery 2018;55:238-246.

19. Khosravi A, Vakhshoori M, Sharif V, Roghani-Dehkordi F, Najafian J, Mansouri A. Comparison of survival rate and complications of percutaneous coronary intervention, coronary artery bypass graft, and medical treatment in patients with left main and/or three vessel diseases. ARYA Atheroscler 2020;16:85-93.

20. Lee K, Ahn JM, Yoon YH et al. Long-Term (10-Year) Outcomes of Stenting or Bypass Surgery for Left Main Coronary Artery Disease in Patients With and Without Diabetes Mellitus. J Am Heart Assoc 2020;9:e015372.

21. Tam DY, Dharma C, Rocha R et al. Long-Term Survival After Surgical or Percutaneous Revascularization in Patients With Diabetes and Multivessel Coronary Disease. J Am Coll Cardiol 2020;76:1153-1164.

22. Huckaby LV, Sultan I, Ferdinand FD et al. Matched Analysis of Surgical Versus Percutaneous Revascularization for Left Main Coronary Disease. Ann Thorac Surg 2021.

23. Hueb W, Lopes N, Gersh BJ et al. Ten-Year Follow-Up Survival of the Medicine, Angioplasty, or Surgery Study (MASS II). Circulation 2010;122:949-957.
